# Complications of psychotherapy for borderline personality disorder: A clinical perspective

**DOI:** 10.1177/10398562251362710

**Published:** 2025-07-29

**Authors:** Beatrice Swan, Stephen Allison, Jeffrey CL Looi, Roger Mulder, Tarun Bastiampillai

**Affiliations:** College of Medicine and Public Health, 1065 Flinders University, Adelaide, SA, Australia; College of Medicine and Public Health, 1065 Flinders University, Adelaide, SA, Australia; Consortium of Australian-Academic Psychiatrists for Independent Policy and Research Analysis (CAPIPRA), Canberra, ACT, Australia; Consortium of Australian-Academic Psychiatrists for Independent Policy and Research Analysis (CAPIPRA), Canberra, ACT, Australia; School of Medicine and Psychology, 2219 The Australian National University, Canberra, ACT, Australia; Department of Psychological Medicine, University of Otago, Christchurch, New Zealand; College of Medicine and Public Health, 1065 Flinders University, Adelaide, SA, Australia; Consortium of Australian-Academic Psychiatrists for Independent Policy and Research Analysis (CAPIPRA), Canberra, ACT, Australia; Department of Psychiatry, Monash University, Clayton, VIC, Australia

**Keywords:** borderline personality disorder, psychotherapy, complications, management

## Abstract

**Objective:**

Psychotherapy is the mainstay of treatment for Borderline Personality Disorder. There are unique benefits for patients through psychotherapy, however there are also complications that are under-recognised and have not been neatly summarised for clinicians and trainees. We present a clinical perspective on the potential complications that may arise from treatment.

**Conclusions:**

Addressing complications is an essential part of high quality and ethical clinical practice. Potential complications of psychotherapy for Borderline Personality Disorder include: escalation of Non-Suicidal Self-Injury (NSSI) and suicidal behaviours, dependency, boundary violations, demoralisation, re-traumatisation, labelling, opportunity cost, and financial issues. Recognition and management of these complications may improve treatment and recovery for patients, and is essential to high quality psychotherapy training and peer review. Further research is needed on the broader challenges and complications associated with non-psychotherapeutic aspects of BPD management.

Borderline Personality Disorder (BPD) is a highly contentious diagnosis in relation to nomenclature, aetiology, clinical presentations, and treatment recommendations.^1^ BPD is characterised by significant impulse-control and emotional dysregulation, resulting in impairment in social, psychological, and occupational functioning. Gunderson, Linehan, and Paris shepherded this diagnosis from conceptualisation and treatment primarily within the psychoanalytic framework towards generalist psychiatric paradigms.^2^ Previously, persistent problems in diagnostic reliability, as well as theoretical bias against personality diagnoses, led to behavioural therapists and general psychiatrists eschewing assessment and treatment intervention for those with BPD.^3^

Despite its complexities, BPD is a very treatable condition. Psychotherapy is the treatment of choice, and multiple psychotherapies have been reported to be effective. In randomised trials of BPD treatment, results indicate that psychotherapy reduces the risks of Non-Suicidal Self-Injury (NSSI) and suicide-related behaviour, and improves psychosocial functioning.^4^ However, there is little consideration of the risk of psychotherapy complications in randomised trials, which reduces their real-world usefulness for clinicians and patients.^5^ We provide an overview of the possible complications encountered by mental health clinicians in the psychological management of BPD. Previously conceptualised as a treatment relatively free of complications, there is increasing evidence of adverse effects associated with psychotherapy. Approximately 10% of people will experience adverse effects in psychotherapy, and this may be higher for those suffering personality disorders.^6^ Adverse effects vary in the degree of physical, psychological, or social suffering, and associated impairment in functioning.^5^ There are also detrimental impacts of treatment in hospital settings outside of psychotherapy, however these will not be addressed in this article. We aim to provide a practical clinical update to address complications of the psychotherapeutic care of those with BPD treatment.

## Complications of psychotherapy for BPD

### Escalation of non-suicidal self-injury (NSSI)

Many people with BPD engage in maladaptive coping behaviours such as Non-Suicidal Self-Injury (NSSI) as a way to ameliorate strong feelings, emphasise unmet emotional pain, and to seek care. These behaviours may be secondary to a relative skills deficit in emotion regulation in the absence of secure attachment. During acute care/hospital/inpatient treatment, a dynamic/shifting workforce and bed pressures to discharge may result in perceived or actual abandonment, which can precipitate an escalation of NSSI.^7^ When a patient engages in NSSI, the clinician may experience helplessness anger engendered by the patient, and may respond in a mis-attuned or invalidating way, inadvertently exacerbating the patient’s feelings of abandonment, and worsening the patient’s mood.^4^

Supervision, team coordination and peer groups may assist clinicians in pre-empting or responding to NSSI escalation. Additionally, many therapists implement pauses in treatment until patients are able to cope and respond in a way that is more adaptive and less harmful, whilst still validating distress. It is important that clinicians help clients to understand the functions of this behaviour and not become overly reactive around escalations in NSSI.

### Dependency/loss of self-management capacity, service over-dependence

In the absence of a secure attachment system, people with BPD are vulnerable to dependent relationships with those that provide professional care. This may precipitate an over-reliance on healthcare services, at the expense of self-management, and secure relationships with others.

Maintaining strong patient-clinician boundaries is necessary to manage dependence and promote autonomy and self-agency. However, this can be challenging as patients necessarily develop a trusting working relationship with the treating clinician, yet are conversely warned against becoming dependent. Additionally, services can, at times, enact punitive approaches under the banner of boundaries. Thus, clinicians must uphold appropriate professional-personal boundaries while engaging effectively through the vicissitudes of psychotherapy. Stepped-care programs have been suggested to counteract over-dependence on clinicians and care services, however, research indicates these have not consistently been implemented.^7^

### Boundary violations

Boundary violations can occur for both people suffering BPD and professional and personal carers. BPD commonly occurs in the context of childhood trauma, leading to attachment issues and difficulties understanding healthy boundaries in relationships. When boundary violations occur, the therapeutic alliance may be insecure or disrupted, which can occasionally lead to termination of treatment and perceived abandonment of the patient; the overall result is worsening of psychological distress. For example, patients may persistently email clinicians outside of hours, resulting in punitive interventions such as cessation of appointments, triggering fears of abandonment and distress. Conversely, treating clinicians can be drawn into violating boundaries, and thus may overstep in caring for their patients. Therefore it is incumbent on clinicians to be aware of the higher risk of boundary violations with BPD, and to try and set clear and consistent boundaries. In addition, it is important for clinicians to understand their own boundaries and to be able to model these in a way that helps the patient to understand what is healthy.

### Demoralisation and despondency due to lack of progress

Mental healthcare professions are prone to expectations of healing, partially through the drive for timely patient improvement.^8^ Unrealistic therapeutic expectations may lead to claims for treatment that are unrealistic or discordant with patients’ psychopathology, the result of which may be perceived lack of progress and demoralisation.^8^ In parallel, there has been a fundamental shift towards consumerism within the doctor-patient relationship, with the patient’s expectation at risk of exceeding realistic treatment outcomes.^9^ Change in psychopathology of BPD, and outcomes, can occur frustratingly slowly for patients and clinicians. Lack of progress may also increase helplessness and self-blame. Acknowledging improvement and recovery can also be threatening for clients. For some, acknowledging improvement can be invalidating, and lead to fears of abandonment or withdrawal of support. Finally, chronic feelings of emptiness are often experienced by people with BPD and may be accompanied by low self-worth. Guilt for perceiving oneself as not able to meet expectations may predispose worsening of low self-worth.^10^ This can lead to demoralisation which becomes an independent risk factor for suicidal ideation and behaviour.^11^

### Re-traumatisation

People with BPD are 14 times more likely to report childhood adversity than non-clinical controls, and three times more likely than other psychiatric patients.^12^ There is an increased baseline risk of re-traumatisation in psychotherapy, through either interpersonal interaction or environmental circumstances. Stigma from a BPD diagnosis may also be re-traumatising, if patients are invalidated or treated in a way that is punitive, reminiscent of adverse childhood experiences.^13^ Establishing a safe therapeutic environment, and being attuned to hyper or hypo-arousal that manifests when re-traumatisation occurs, may assist in managing this complication.

### Introspection and labelling

Attachment disturbances may adversely impact a person’s ability to understand themselves, and how they interact with the world. This has implications in the development of self-identity.^14^ There is potential for pathologisation of normal human behaviour, through labelling, to occur due to an unstable sense of self paired with over-introspection. In the context of greater mental health literacy and increasing presence of the language of mental health within social media networks, there is the risk that non-specific distress and low psychological well-being is channelled into diagnostic categories.^9^ This may be particularly harmful during adolescence, in which intense emotionality, unstable relationships, impulsivity, difficulties with self-concept, and exploratory and risk behaviours form part of normal adolescent experiences.^12^ Pathologisation of normal experiences may lead to excessive help-seeking and pursuit of treatment rather than more appropriate social or vocational activities.

### Opportunity cost

BPD has a high burden of disease. The focus of existing interventions has largely been on managing clinical symptoms via structured psychotherapies with limited impact on key domains of functional recovery such as social connectedness and employment.^1,15^ The opportunity cost of therapeutic treatment engagement may prevent the harnessing of positive changes in social circumstances,^16^ or vocational training or employment. People with BPD attain fewer educational qualifications and experience higher rates of unemployment. Therapeutic engagement, if at the cost of vocational or educational opportunities, may perpetuate this challenge.^14^

### Equity issues

Psychological treatments for BPD are mostly limited to face-to-face, individual, or group therapies, many of which have historically been technically complex and required lengthy training. However, there is growing evidence that more generalist approaches, such as Good Psychiatric Management (GPM), have benefit for patients, and may be less resource-intensive.^17^ Yet, many mental health professionals still believe that BPD-specific psychotherapy is esoteric, leading to a lack of willingness and confidence in treating patients. Thus, services that are able to provide access to clinicians who are confident in and willing to treat BPD, with access to regular supervision, are limited, due to workforce capacity constraints.^15^ People with BPD may need to access partly self-funded treatment for their symptoms, such as private GPs, psychologists and psychiatrists, due to a lack of more heavily subsidised or free options. Psychological therapy may only be accessible to those who have sufficient means, leading to inequity for those without.

## Conclusions

Despite its efficacy, complications can arise during psychotherapy for patients with BPD. These complications have historically been underplayed, with potential for iatrogenic harm during treatment for patients. Screening for complications during therapy may help with identification and management of these problems and should be incorporated in psychotherapy training, clinical practice, and peer review. Psychotherapeutic complications must be considered, and strategies to mitigate complications employed, based on the limited evidence to date. However, there is a need for more detailed studies of the adverse effects of psychotherapy to provide guidance for interventions, which will in turn need to be evaluated for efficacy and complications.^18^ Other, non-psychotherapeutic complications of BPD treatment warrant separate research that encompasses evaluation of efficacy, adverse effects and interventions. Better understanding of the management of psychotherapeutic complications will facilitate more effective care that improves the quality of life for those with BPD and, if well managed, these complications may lead to insights for both patient and therapist.
